# Optimizing data collection for obstetrical ultrasound research at the primary health care level in rural Uganda

**DOI:** 10.1080/16549716.2024.2436715

**Published:** 2025-01-17

**Authors:** Renny Ssembatya, Abimbola Leslie, Kristen DeStigter, Erika M. Edwards, Joyce Nayiga, Samalie Nakibirango, Julian P. Arapa, Nicholas Bahati, Blandina Busingye, Thomas Eremu, Mary Nakafeero, Micheal F. Ssenoga, Frank B. Williams, Delia Horn

**Affiliations:** a Imaging the World Africa, Kampala, Uganda; b Department of Radiology, University of Vermont Medical Center, Burlington, USA; c Department of Radiology, The University of Vermont, Burlington, USA; d Mathematics and Statistics, Pediatrics, The University of Vermont, Burlington, USA; e Department of Radiology and Radiotherapy, Imaging the World Consortium for Health Limited, Makerere University College of Health Sciences, Kampala, Uganda; f Imaging the World Consortium for Health Limited, Kampala, Uganda; g Data Clerk, West Nile, Uganda; h Data Clerk, Western Uganda, Uganda; i School of Public Health, Makerere University, Kampala, Uganda; j Imaging the World Center for Health and Innovation, Kampala, Uganda; k Department of Obstetrics and Gynecology, Ochsner Baptist Medical Center, New Orleans, USA; l Pediatrics, The University of Vermont, University of Vermont Medical Center, Burlington, VT, USA

**Keywords:** Imaging the World, Imaging the World Africa, low- and middle-income countries, prenatal ultrasound, neonatal mortality, maternal mortality, data accuracy, data completeness

## Abstract

**Background:**

Neonatal and maternal mortality remains high in low- and middle-income countries (LMIC), especially in sub-Saharan Africa. Quality data collection is crucial to understand the magnitude of these problems and to measure the impact of interventions aimed at improving neonatal and maternal mortality. However, data collection in the low-income country setting, especially in rural areas, has been a challenge for researchers, policy makers, and public health officials. Here, we describe the methodology, experience and lessons learned while collecting data at lower-level primary health care facilities in rural Uganda.

**Methods:**

Data collection was performed at Health Center III sites in rural Uganda, in partnership with Imaging the World and its affiliate Imaging the World Africa. The primary purpose of the data collection was to study the efficacy and clinical effect of introducing prenatal ultrasound services at these sites. Local data clerks were hired to perform the data collection through a combination of intensive training and on-the-ground support. Frequent oversight was used to support data collection.

**Results:**

Of 2,397 enrolled pregnant women, 1,977 (82.5%) had complete outcome data. Upon independent expert audit, the data were >80% accurate for 10/11 variables and >90% accurate for 6/11 variables. Overall, the data collected at the rural HCs were 90% accurate.

**Discussion:**

Accurate and complete data collection is possible in an LMIC setting if appropriate training and oversight are employed.

## Paper Context

- **Main findings:** High-quality data collection is possible in the rural low- and middle-income country (LMIC) setting using locally trained Data Clerks.
- **Added knowledge:** Clear, detailed methods for obtaining high-quality data in the rural LMIC setting are described.
- **Global health impact for policy and action:** Describing methods for high-quality data collection in rural LMIC will allow for more complete and accurate data to be collected in this setting, promoting understanding of the health needs and successes of those living in rural LMIC globally.

## Background

Neonatal and maternal mortality surrounding childbirth remains high in low- and middle-income countries (LMIC). Nearly 300,000 women die annually from preventable complications [1,2]. Since most rural communities in sub-Saharan Africa lack the basic diagnostic capacity of ultrasound, obstetric problems like twins, breech, placental abnormalities and pregnancy dating issues go unidentified and unaddressed [3,4]. The high perinatal mortality and morbidity in LMIC is in large part due to a lack of access to resources that are considered standard of care during pregnancy in high-income countries [5,6]. Ultrasound can diagnose complications that result in maternal morbidity and mortality, yet it is underutilized because traditional ultrasound requires a specialist to scan and make clinical management decisions at the point of care [5].

The World Health Organization recommends at least one ultrasound scan before 24 weeks of gestation to estimate gestational age, improve detection of fetal anomalies and multiple pregnancies, reduce induction of labor for post-term pregnancy, and improve a woman’s pregnancy experience [7]. A 2022 update translates the recommendation into contextually appropriate guidelines for routine antenatal care (ANC) at the country level, still with the recommendation for scanning before 24 weeks [8]. For the past 15 years, Imaging the World (ITW) and its affiliate non-governmental organization (NGO) Imaging the World Africa (ITWA) have implemented a ‘basic obstetric ultrasound package’ in rural Uganda Health Center III sites (HCIII) that includes fit-for-purpose portable ultrasound equipment, training, peer-review Quality Assurance, and continuous supervision via teleradiology. Operating in three countries, ITW/ITWA have developed a scalable model to improve access to basic obstetric ultrasound in the rural LMIC setting [9–11]. Based on the years of anecdotal observations at over 30 rural HCIII in Uganda, ITW/ITWA set out to validate the evidence basis for point-of-care obstetric ultrasound in the third trimester, focusing on diagnosis and management of complications (‘screen and refer’) in existing hub and spoke health systems.

**Figure 1.**
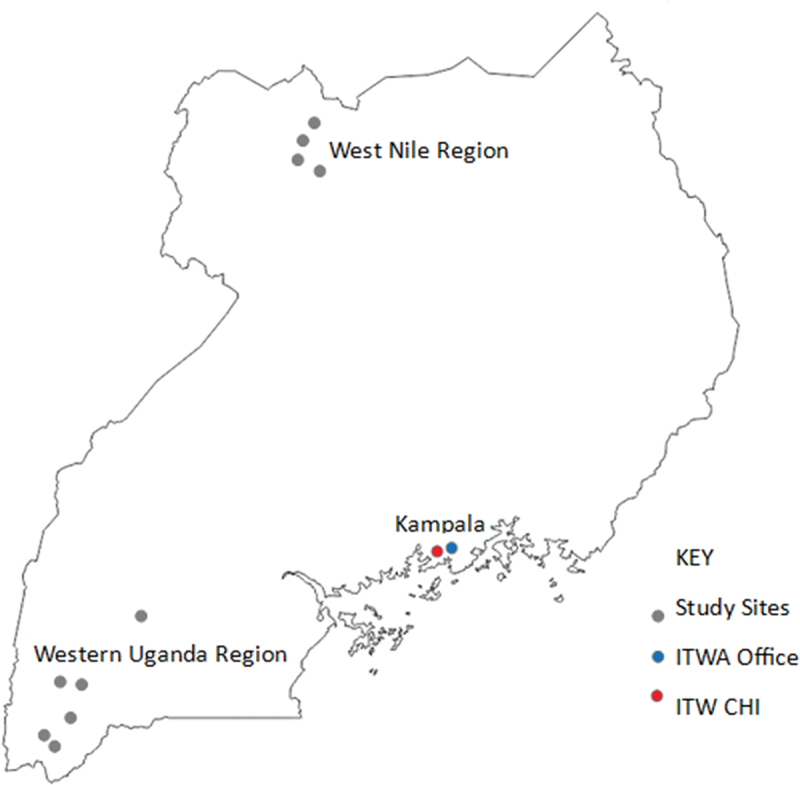
Study sites.

This study leveraged an existing African Academy of Sciences Grand Challenges Africa [12] grant geared to expand the ITW/ITWA ultrasound program to seven ultrasound-naive HCIII in rural Western Uganda and the Northern region, including the refugee settlement of West Nile region. This was done in partnership with the Office of the Prime Minister and Medical Teams International (Figure 1).

Research was supervised under a protocol approved by Institutional Review Board (IRB)/Uganda National Council for Science and Technology (UNCST) to determine the effect of the point-of-care obstetric ultrasound intervention on clinical management, referral patterns, and neonatal and maternal outcomes.

From mid-June 2022 to September 2023, longitudinal prospective data were collected on patients during the third trimester of pregnancy (28–41 weeks). Data included demographic information, past medical and obstetric history, prenatal care during the index pregnancy, ultrasound findings (where applicable), referral recommendations, and health outcome metrics for mother and baby surrounding labor and delivery. Following focused classroom training as well as remote and on-site supervision, we utilized on-site ‘Data Clerks’ (DC) at each HCIII facility for the data collection. To our knowledge, no other study has employed DC for longitudinal collection of obstetric ultrasound data at the LMIC primary health care level. During this process, it became clear that the experiential learning of the data collection itself was of value. With increasing interest in basic point-of-care obstetric ultrasound, including extensive funding of Artificial Intelligence-enabled technologies, improved data collection at rural HCIII in LMIC will be critical to show meaningful clinical implementation. This manuscript details our data collection experience and suggests practical actions to increase the likelihood of capturing quality data in LMIC.

## Methods

**Figure 2.**
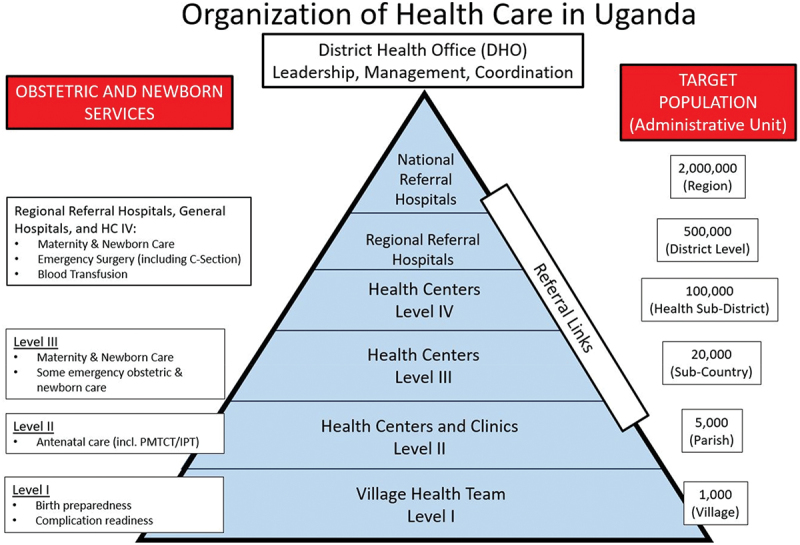
Organization of health care in Uganda – obstetric and newborn services. Adapted from Northern Uganda Health Integration to Enhance Services Assessment Report for Emergency Obstetric Care in Northern Uganda, 2014. Abbreviations: C-section, cesarean section; HC, health center; IPT, intermittent preventive treatment; PMTCT, prevention of mother-to-child transmission (of human immunodeficiency virus).

Data collection was performed from mid-June 2022 through September 2023 at the HCIII (Figure 2) level in rural communities where pregnant women accessed care. We collected data at a total of 10 hCIII facilities in three districts of Western Uganda and one district in West Nile region. The West Nile sites border South Sudan and serve as the largest refugee population in sub-Saharan Africa. Our intervention at these public governmental facilities was facilitated through formal partnerships with the Uganda Office of the Prime Minister and Medical Teams International. Ultrasound services at the private facilities in Western Uganda were implemented in formal partnership with the Uganda Catholic Medical Bureau, which delivers approximately 50% of all health care in the country [13]. Patients or the public were not involved in the design, or conduct, or reporting, or dissemination plans of our research.

For those living in the rural parts of the country, the HCIII offers the majority of primary care services. It is the most accessible level of the health system that has routine obstetrical care available, and so is where most women access prenatal care and labor and delivery services. There are no cesarean section surgeries available at HCIII, so complications identified at this level are referred to HC IV, district, or regional referral hospital facilities. Each HCIII serves a population of approximately 20,000 people [14]. Here, we describe the novel approach we took to collecting longitudinal data in this rural LMIC setting.

### Preparation

The project received IRB approval in the United States at the University of Vermont (STUDY00002441) and in Uganda at Mengo Hospital (study No. MH2021-17) and the UNCST (Registration No. HS1994ES). All DC consented to have the data accuracy and completeness and data collection methods published after the initiation of the study, once it was determined that sharing these data collection methods could be of benefit to the scientific community. Study data (pre-loaded Case Report Forms, CRF) were collected and managed using REDCap (Research Electronic Data Capture) electronic data capture tools hosted at the University of Vermont [15,16]. REDCap is a secure, web-based software platform designed to support data capture for research studies. Cost-effectiveness and the ability to download projects and collect data offline were important to our use of this tool, especially in rural Uganda where access to internet services can be severely limited.

The study team hired and trained DC to perform data collection at each of the 10 clinical sites. The overall aim of this training was to equip local individuals with the knowledge and skills they needed to collect accurate and complete data for the obstetric ultrasound study. One DC was hired per clinical site from the local community, so they would be familiar with local languages and dialects, customs, and live within relative proximity to the clinic. Baseline competency was assessed through a standardized interview process. Required DC attributes were local tribal language fluency, ability to travel to local HCIII daily for full-time work, background experience in health care, baseline technological literacy (Microsoft Excel and Word), and baseline medical literacy (knowledge of/experience with medical records management).

DC were paid fair market value less benefits and taxes at a total cost of $24,000, or 7% of the study budget. This amount did not include the cost of their supplies, supervision by other study team members, monitoring and evaluation audits of their work, or the statistician to review their data. Before data collection began, all DC were transported to the Imaging the World Center for Health and Innovation in Kampala, Uganda, for a 4-day comprehensive training in REDCap data collection specific to this project. They also underwent International Council for Harmonization of Technical Requirements for Pharmaceuticals for Human Use guideline for good clinical practice research training. They were each issued a tablet with study REDCap data collection project software downloaded.

The curriculum was delivered in two phases. Phase 1 covered International Council for Harmonization of Technical Requirements for Pharmaceuticals for Human Use guideline for good clinical practice research training and Human Subjects Protection. Phase 2 focused on data collection specific to this study (Table 1).

**Table 1.** Data clerks training curriculum.

Phase 1: Ethics in Research	Phase 2: Women’s Health, Obstetrics, and Newborn Care
Historical evolution of ethics in clinical research	Medical terminology
Primary guidelines of ethical considerations in clinical research	Common obstetric diagnoses
Key elements of the primary guidelines in ethical clinical research	The Ugandan Maternal (antenatal care) Passport
Importance of ethics in clinical research	Ugandan Antenatal and Maternity Register
Consequences of unethical conduct in clinical trials and resolution of issues pertaining to actual or potential unethical conduct	Overview of Uganda’s Referral System
Research subject privacy	Study workflow
Basic principles of bioethics and the informed consent form	Study Case Report Form (CRF) and data collection application (REDCap, Research Electronic Data Capture)

At the end of the training, the DC were expected to (i) have a basic understanding of the study flow, data sources, and study procedures, (ii) understand the CRF to ensure that all relevant data were collected to the highest level of quality possible, and (iii) have a clear understanding of research ethics to ensure that the rights of research participants were upheld throughout the data collection process. All DC scored at least 85% in practical and written assessments at the completion of the course.

Patient data were collected from various sources for this study, including direct interviews with patients coming for prenatal visits or ultrasounds, interviews with mothers bringing infants for immunizations, review of the Ministry of Health (MOH) Maternal Passport and Maternity Register at the HCIII, discussion with nurses and midwives at the HCIII, chart review at the regional referral hospitals, information collected via Village Health Teams (VHT), and direct interviews with patients via postnatal phone call. The MOH Maternal Passport and the MOH Maternity Register are health management information systems (HIMS) employed by the MOH in Uganda for collecting and storing crucial patient data particularly in the rural setting [17]. VHT are made up of community volunteers selected by the community to provide correct health information and act as links to health services. VHT are trained with a basic MOH health promotion package as well as add-on modules as indicated. They are also trained to do very basic health assessments of pregnant women and children, provide immunizations, provide very basic antenatal and newborn care, and give first aid [18].

Pregnant women (‘mothers’) were followed from their first prenatal visit in the third trimester at 28–41 weeks, through 42 days post-partum. Data were collected via the following process: The DC collected data from mothers at the ANC visit, cross-referenced the data with the midwife, maternity register, and maternal passport, and entered the data into REDCap. The DC followed up with the mother, midwives, and maternity register at or shortly after the time of delivery. If the mother delivered elsewhere than HCIII, the DC attempted to call the mother and the referral hospital and followed up with the VHT. The DC then entered the final outcomes data in REDCap.

**Figure 3.**
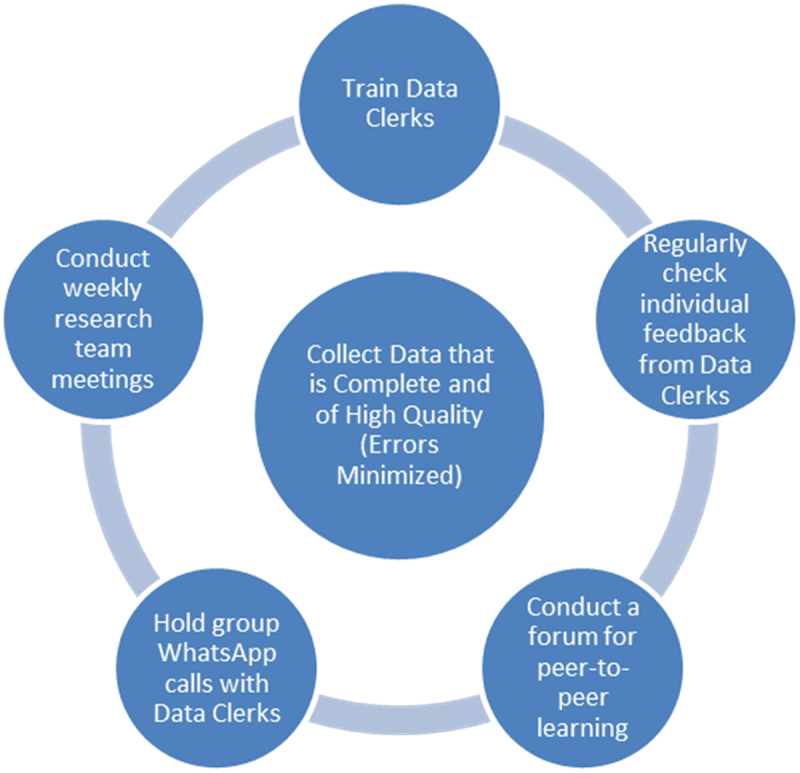
Process to improve data quality at HCIII.

Throughout the 1.25-year data collection period, various methods were employed to ensure ongoing high-quality data collection (Figure 3).

### Meetings

Throughout the study period, the study team (members in Uganda and the United States) met virtually on a biweekly basis, or weekly if concerns or events arose. Notes were taken on any common errors that were identified as well as outliers in the data that were flagged as incorrect. Feedback was given to DC directly via individual WhatsApp calls following these meetings to correct these errors.

Additional quarterly online group calls were held over WhatsApp with the study team (Horn, Leslie, Ssembatya) and all the DC. Initially, these calls focused on simple data collection errors and explaining data collection fields that were causing confusion. Later, these became a source for conversations around strategies for preventing loss to follow-up, and a forum to discuss any challenges the DC were facing. Overall, both site-specific and general challenges to data collection were identified. Knowledge was documented for incorporation into a manual for future training.

Weekly status reports were given to individual DC, via phone calls and document sharing on WhatsApp. A platform for daily phone calls was created to enable two-way communication between the monitoring and evaluation team at ITWA in Kampala and the DC. The information technology manager at ITWA provided remote troubleshooting to DC who had issues with their tablets and laptops.

Quarterly field support supervision visits were made by the ITWA team (including the research coordinator, IT lead, and logistics lead, as well as ultrasound trainers) to support the data collection process and provide continuing professional development courses to the health workers who were doing scans. Concerns aired during these phone calls or site visits were shared with the research team to collectively devise solutions to these issues.

Finally, additional one-on-one follow-up calls with DC were held during the study period, to provide direct feedback on the completeness and accuracy of their data collection, and to discuss any challenges they were facing. These occurred over WhatsApp.

### Data errors

Data collection errors were grouped into the following categories: non-response error; response error (respondents intentionally or accidentally providing inaccurate responses); interviewer error (interviewers incorrectly record information; were not neutral or objective; influence the respondent to answer in a particular way; or assume responses based on appearance or other characteristics); and processing error (errors that occur in the process of data collection, data entry, coding, editing and output). The errors observed and the solutions applied to reduce them were categorized by these types (Table 2).

**Table 2.** Error types and solutions.

Error Type	Errors Observed	Solution
Non-response error	● Outcomes at 28 days (neonatal) and 42 days (maternal) not obtained ● Obstetric history left blank ● Mothers “lost to follow-up” – lack of phone access, returned to home country (refugee), delivered at home, delivered in a different district from where they sought antenatal care	● Obtain more than one contact number per mother ● Contact mother 2 weeks prior to expected delivery to ensure accurate contact ● Follow-up with mothers when they return to clinic for their child’s immunizations ● Try to reach mother through neighbors or VHT
Response error	● No report of prior abortions (miscarriages) ● Misunderstood question about self-treating for malaria	● Clarify confusing questions discussed in WhatsApp calls ● Clarify meaning of medical “abortion”
Interviewer error	● Fewer measures taken to obtain outcomes from refugee mothers ● Follow-up data for mothers who delivered on the weekend often not pursued	● Support reaching refugee mothers provided through VHT ● Collaboration tools (hand-off process) among nurses/midwives and DCs to obtain data from weekend deliveries
Processing error	● Lack of translators in refugee sites ● REDCap, tablet, or other information technology issues ● Hemoglobin entered incorrectly ● Dates entered incorrectly	● Coordinate collection with VHT and better community engagement ● Support from ITWA information technology lead, with more regular site visits ● Hemoglobin entry mode in REDCap changed ● Erroneous dates identified and cross-referenced with Maternal Passport and MOH Registe

Correct means the variable entered by the DC in REDCap matched the records at the HCIII on independent audit. Incorrect means the variable entered in REDCap did not match the HCIII records.

A patient ‘loss to follow-up’ checklist identifying patients who failed to return was developed and distributed to all DC via WhatsApp. DC used this to document any study participant that they felt was lost to follow-up 4 months post-delivery. This checklist included marking with confirmation that all the strategies provided for preventing loss to follow-up had been employed (Table 4). At the time, the checklist was distributed, individual WhatsApp phone calls were held with each DC to explain the checklist and to inform them that all DC would be ranked based on the completeness of their outcome data, to incentivize them to obtain as complete outcome data as possible. Through this study’s process to improve data quality (Figure 3), specific problems with individual DC were identified and investigators employed focused remediation with good success.

Investigators retained 8 of 10 DC from initial recruitment through the term of the study. One DC gave prior notification of her intention to leave, and she mentored a replacement who was also given additional training (on-site training in the DC curriculum) during quarterly routine support supervision visits by the ITWA team. Another DC had consistently poor performance with low numbers of enrollment, inaccurate data, and few complete outcomes, and so after many individual calls and attempts at remediation, this individual’s employment was terminated, and another already-trained local DC took over that site (responsible for two sites). By this time, these sites were not enrolling new study participants and were following up only existing participants for birth outcomes.

An independent audit of the accuracy of this data was performed after data collection concluded (Makerere University School of Public Health). This audit compared study data with data found in maternity logs at the HCIII sites for a preset number of variables to determine its accuracy.

## Results

The birth outcome data were very much complete, with 5/10 sites having >90% complete data and 10/10 sites having >70% complete data (Table 3). Overall, 1,977 of 2,397 (82.5%) had complete prenatal and birth outcome data. The data were >80% accurate for 10/11 variables audited and >90% accurate for 6/11 variables audited. Overall, the data were 90% accurate (Table 4).

**Table 3.** Data completeness by site.

Health Center	No.	%
HC I (*n* = 312)	246	78.9
HC II (*n* = 90)	65	72.2
HC III (*n* = 94)	90	95.7
HC IV (*n* = 312)	288	92.3
HC V (*n* = 207)	159	76.8
HC VI (*n* = 375)	321	85.6
HC VII (*n* = 118)	109	92.4
HC VIII (*n* = 176)	23	86.9
HC IX (*n* = 472)	458	97.0
HC X (*n* = 241)	237	98.3

**Table 4.** Data accuracy by variable.

Variable	Correct	Incorrect	Total	% Correct
Prior Surgery	206	16	222	93
Prior C-Section	204	18	222	92
Referral to a Higher Level of Care	182	39	221	82
Birth Method	197	24	221	89
Neonatal Mortality	409	62	471	87
Intrauterine Pregnancy	184	12	196	94
Number of Fetuses	184	5	189	97
Anterior Placenta	151	38	189	80
Posterior Placenta	149	40	189	79
Low Lying Placenta	187	2	189	99
Partial Previa	188	1	189	99

DC were ranked based on their ability to obtain complete outcome data from enrolled study participants. This system was implemented partway through data collection in order to incentivize complete data collection. Gold indicates they acquired complete outcome data from >95% of enrolled participants, silver means they achieved this for 85–95% of enrolled participants, and bronze means they obtained complete outcome data for <85% of the study participants they enrolled. Overall, 40% (4/10) of DC achieved gold level, 40% (4/10) achieved silver level, and 20% (2/10) achieved bronze. In one instance, however, the DC who replaced a poorly performing DC was given gold designation for ‘extraordinary effort’ in acquiring data from mothers who had been previously enrolled. All DC were presented with a certificate acknowledging their level of achievement. Gold-Level DC were included in the analysis and interpretation of our results and revision of intellectual content, and therefore are included as authors on this paper.

### Types of data errors

Data errors fell into distinct types (Table 4). Early on, non-response errors and response errors – errors due to DC not understanding questions or leaving fields blank – were the most common. These errors were easily remediated during WhatsApp calls, and DC quickly became comfortable understanding data fields. However, there were issues with interviewer error throughout the data collection process, in that DC had differing approaches and expended variable amounts of energy for obtaining follow-up data from mothers who had already delivered. Data collection generally improved over the course of the study, as DC shared ‘best practice’ methods with their peers who were struggling. Universal improvement in the DC effort placed into obtaining follow-up data was seen when the Gold/Silver/Bronze ranking system was introduced.

## Discussion

To the investigators’ knowledge, no other study has employed DC for longitudinal collection of obstetric ultrasound data at the LMIC primary health care level. There are many efforts to improve reproductive, maternal, newborn, and child health in LMIC. However, research studies assessing technology or practices to reduce morbidity and mortality are hindered by challenges with accurate data collection in this setting, especially in rural areas. Stevenson et al. described the importance of data collection in resource limited settings in 2021, stating ‘without data, health systems are powerless to improve the care they provide, learn from their own practice, collaborate effectively with other centers, or advocate successfully for the resources they need. In LMIC, where data are scarce and often of poor quality, there is frequently a large disconnect between providers on the ground and the political bodies directing funds. Quality data can support clinicians and clinical managers to prioritize certain actions’. The authors stated that ‘to elucidate information about quality of care and system performance surrounding the time of birth, input and process metrics that relate to the mortality targets and high-impact interventions associated with reduced perinatal mortality rates are recommended’ [19].

This study shows that accurate and complete data collection at the primary care level (HCIII) in the LMIC setting is possible. The key to this success was engaging, training, and retaining local individuals to perform the data collection, as their familiarity with the study sites and the surrounding community, and their ability to interface with study subjects, was of critical importance. Very few DC were lost to attrition as strenuous travel was not involved. Anecdotally, DC became engaged in the system of care, collaborating with midwives to improve workflow efficiency. By providing data collection skills and training, as well as incentivizing DC with certificates for outstanding achievement, the DC were engaged and motivated beyond simple monetary remuneration.

There were various strengths and limitations to this study. This study demonstrates that complete and accurate data can be collected in the rural LMIC setting, and it describes successful data collection in the rural LMIC setting in sufficient detail that it can be replicated. However, while highly complete and accurate for the setting, data collection could be further improved. Finally, this is a description of data collection in one specific rural LMIC setting and may not be generalizable to other rural LMIC settings with differently structured health care systems.

### Lessons learned

There were several lessons learned from this work. Foremost, the study team, including the DC, underestimated how difficult it would be to get follow-up data from mothers, particularly those living in refugee settlements. This patient follow-up was critical to quality data collection. In the end, multiple methods were used to contact women, including phone numbers of friends and family members. Employing the local VHT proved very useful in cases of suspected ‘loss to follow-up.’ For future studies, the authors would recommend involving VHT at the beginning of the study rather than when collecting outcome data. The VHT can personally locate mothers in lieu of collecting telephone numbers, due to their close proximity and geographical knowledge of the communities. Women were more likely to follow-up if all the study remuneration was not provided at their initial intake. Study participants were paid for their participation, as is the national standard in Uganda. Remuneration was distributed in three parts over the course of their participation in the study − 1/3 at study entry, 1/3 after delivery, and 1/3 after final outcome data had been collected. This change was made 4 months into the study when the team realized that mothers who were not vested from the start often would not return to the HCIII for further services.

The authors would recommend including local health center midwives and DC in the design of the data CRF. This would allow end-users to provide a perspective on fields or questions that may be confusing. It was beneficial to use REDCap capabilities such as input masks or conditional and skip logic to force the format of answers (rather than free text). The CRF should be limited to variables of paramount interest, using binary answers whenever possible to reduce confusion. The authors recommend formal, structured training of new DC following attrition, as well as a standardized written and verbal assessment of DC following training, prior to the start of data entry to ensure readiness, and quarterly to identify issues requiring focused remediation. Additionally, the authors recommend field pre-testing of data collection tools, including the mobile data collection device, prior to study initiation. Finally, the authors recommend formal data audits be performed quarterly throughout the study.

### Future directions

The data collection described here was specific to this study. However, ITW/ITWA will continue to use DC to collect data at rural primary care facilities throughout Uganda, Malawi, and Kenya. Evidence-based outcomes will be used to optimize care delivery and to explore ways to improve maternal and neonatal mortality through point-of-care obstetric ultrasound services. Quality data are of key importance to policy makers who must make strategic and budgetary decisions based on their value to the system. Quality data will support collaborative research with academia and industry, including for Artificial Intelligence-enabled obstetric ultrasound interventions that could further improve access to care and maternal and child health outcomes [20].

## Conclusion

This study shows that accurate and complete data collection at the primary care level in the rural LMIC setting is possible. Partnering with local individuals and organizations is key to this success.
